# Trabecular bone scores in young HIV-infected men: a matched case-control study

**DOI:** 10.1186/s12891-020-3092-0

**Published:** 2020-02-10

**Authors:** Youn Jeong Kim, Kwi Young Kang, Juyoung Shin, Yoonhee Jun, Sang Il Kim, Yang Ree Kim

**Affiliations:** 1 Division of Infectious Disease, Department of Internal Medicine, College of Medicine, The Catholic University of Korea, Seoul, South Korea; 2 Department of Internal Medicine, Incheon St. Mary’s Hospital, College of Medicine, The Catholic University of Korea, #56, Donsu-Ro, Bupyung-Gu, Incheon, South Korea; 3 Division of Rheumatology, Department of Internal Medicine, College of Medicine, The Catholic University of Korea, Seoul, South Korea; 4 Health Promotion Center, Seoul St. Mary’s Hospital, College of Medicine, The Catholic University of Korea, Seoul, South Korea; 5 Division of Endocrinology and Metabolism, Department of Internal Medicine, College of Medicine, The Catholic University of Korea, Seoul, South Korea; 6 Department of Internal Medicine, Seoul St. Mary’s Hospital, College of Medicine, The Catholic University of Korea, 505 Banpodong, Seochogu, 137-701 Seoul, Republic of Korea; 7 Department of Internal Medicine, Uijeongbu St. Mary’s Hospital, College of Medicine, The Catholic University of Korea, Chonboro 271, Uijeongbu, South Korea

**Keywords:** Bone microarchitecture, HIV, Trabecular bone score

## Abstract

**Background:**

Screening for osteoporosis with dual-energy X-ray absorptiometry (DXA) is recommended for male HIV-infected patients only above the age of 50. Recently, trabecular bone score (TBS) has been introduced as a novel tool to assess bone microarchitecture using DXA of the lumbar spine. Few studies have reported TBS values in HIV-infected individuals younger than 50 years of age. This study compared TBS values in young males infected with HIV and matched controls, and investigated the associations between TBS and demographic parameters, clinical parameters, and bone mineral density (BMD) scores.

**Methods:**

A cross-sectional study of BMD and TBS in HIV-infected men (*n* = 80) aged between 18 and 50 years and age- and sex-matched controls (*n* = 80) was conducted.

**Results:**

The proportion of patients with low BMD (Z-score ≤ − 2) was significantly greater among HIV-infected patients than among matched controls (21.3% [17/80] vs. 8.8% [7/80], *p* = 0.027). Mean TBS values were significantly lower in HIV-infected patients than in controls (1.41 ± 0.07 vs. 1.45 ± 0.07, *p* = 0.008). In both groups, TBS values were positively correlated with BMD at the lumbar spine, femoral neck, and total hip (*p* < 0.001); however, TBS was not correlated with body mass index. In the HIV group, TBS was negatively correlated with the duration of tenofovir disoproxil fumarate(TDF) exposure (*p* = 0.04).

**Conclusion:**

Young men infected with HIV had abnormal bone trabecular microarchitecture, as assessed by both TBS and BMD. TBS values were correlated with both BMD and the duration of TDF exposure.

## Background

Advances in antiretroviral therapy (ART) have improved life expectancy dramatically among people living with HIV. However, this improvement gives to rise to concerns about non-AIDS-related comorbidities that may be related to ART and age, such as cardiovascular, renal, metabolic, and bone disease. A meta-analysis showed that the prevalence of osteopenia or osteoporosis is higher in HIV-infected patients than in controls [1]. The pathogenesis of bone loss in HIV-infected individuals is a complex and multifactorial process, with HIV itself, the use of antiretroviral agents, hypogonadism in men, menopause in women, low body mass index (BMI), aging, malnutrition, steroid use, and smoking all associated with bone disease [2]. Hormonal changes in postmenopausal women or in elderly people are associated changes in the bone remodeling cycle, which leads to bone fragility and an increased risk of bone fracture [3, 4].

Bone mineral density (BMD) is determined by the peak bone mass and amount of bone loss and is the standard measure used for the diagnosis of osteoporosis. BMD evaluates bone quantity, rather than bone microarchitecture or composition [5]. Recently, the trabecular bone score (TBS) has been introduced as a novel tool for assessing bone microarchitecture. TBS values are obtained from dual-energy X-ray absorptiometry (DXA) scans of the lumbar spine using a proprietary software program; TBS is a noninvasive, indirect measurement calculated from the projection of the three-dimensional bone structure onto a two-dimensional plane [6]. TBS has been validated as a good prognostic tool for assessing trabecular microstructure independent of BMD and has been reported to increase the accuracy of fracture prediction in patients with BMD above the osteoporotic threshold [7, 8]. However, few studies have evaluated TBS in HIV-infected individuals, especially young male patients. We calculated TBS values from DXA images of the lumbar spine taken from HIV-infected male patients under 50 years of age. The etiology of osteoporosis in HIV-infected patients is multifactorial; therefore this descriptive study included only male HIV infected patients aged less than 50 years to minimize the influence of traditional risk factors associated with low BMD, such as age and postmenopausal status in women. We compared TBS values obtained from HIV-infected young male patients with those from matched healthy controls and investigated the associations between TBS and demographic parameters, clinical parameters, and BMD scores for the lumbar spine and femoral neck.

## Methods

### Study population

Male HIV-infected patients aged between 18 and 50 years were recruited from tertiary university hospitals in Korea from March 2015 to December 2018 for inclusion in this cross-sectional study. Only men were included to avoid potential confounds related to menopause-induced osteoporosis in women. Eighty HIV-infected patients were enrolled. Among subjects who attended routine health check-up examinations in tertiary university hospitals during the same period, controls matched for age and sex were included after BMD data was blinded. HIV-infected patients and control subjects with thyroid disorders, parathyroid malignancies, chronic liver disease, or rheumatoid arthritis were excluded. None of the HIV patients or control subjects took corticosteroids, calcium, or bisphosphonate agents. BMI was calculated as weight divided by height squared (kg/m^2^).

### Clinical and laboratory evaluation

Baseline study visits involved collection of demographic, socioeconomic, and clinical information, and renal profile, liver function tests, and bone profiles, assessment of parathyroid hormone (PTH) and serum 25-hydroxy vitamin D (25[OH]D) levels, and measurement of the bone turnover markers serum C-terminal cross-linking telopeptide of type I collagen(CTX) and osteocalcin. CD4+ T-cell counts and HIV-1 RNA were obtained for subjects with HIV.

### Measurement of BMD and TBS

TBS and BMD were evaluated in all HIV-infected patients and matched control subjects at the time of enrollment. BMD of the lumbar spine (L1–L4) and left hip was measured with the use of DXA scan (Lumbar Prodigy densitometer, Madison, WI, USA). Grams per square centimeter (g/cm^2^), as well as T score (compared with values of young adults of the same sex) and *Z* score (compared with values of adults of the same age and same sex) is reported for the AP lumbar spine and left hip. Low BMD for individuals under 50 years of age was defined as a Z-score ≤ − 2.0 according to the guideline [9]. We evaluated TBS at L1-L4, derived from DXA files from the database using TBS iNsight version 2.1 (Med-Imaps, Pessac, France) as our previous published data [10, 11]. TBS was Control subjects and HIV patients were divided into three TBS groups according to risk of fracture, as described in a recently published study [12]. A TBS score above 1.31 is considered normal (low risk fracture); a value between 1.23 and 1.31 denotes partially degraded bone microarchitecture (interemediate risk frcature); and a value below 1.23 denotes strongly degraded bone microarchitecture(high risk fracutre) [13].

### Ethics

The study was approved by the ethics committee of Seoul St. Mary’s Hospital (study number: KC14OISI0768). Written informed consent according to the Declaration of Helsinki was obtained from all study subjects.

### Statistical analysis

Statistical analyses were performed in SPSS (version 14.0; SPSS Inc., Chicago, IL, USA). Continuous data are expressed as the mean ± SD, and categorical data are expressed as percentages. Clinical variables were compared using an independent t-test, and categorical variables were compared using a Chi-squared test. Spearman’s correlation coefficient was used to analyze the correlations between variables. All tests were two-tailed, and *p*-values < 0.05 were considered statistically significant.

## Results

### Demographic characteristics

The demographic and laboratory characteristics of 80 HIV-infected patients and 80 age- and sex-matched controls are shown in Table 1. In both groups, the mean age of the patients was 39 ± 8 years, and all were male. The two groups did not differ in terms of smoking (*p* = 0.08), alcohol consumption (*p* = 1.0), or presence of diabetes mellitus (*p* = 0.24) or hypertension (*p* = 0.12). BMI was significantly lower in HIV-infected patients than in the control group (22.9 ± 3.0 vs. 24.9 ± 3.3, *p* < 0.001). HIV-infected patients had a greater history of previous fractures (12.5% [*n* = 10] vs. 1% [*n* = 1], *p* = 0.009) and presence of dyslipidemia (10.0% [*n* = 10] vs. 0%, *p* = 0.028).

**Table 1:** The comparison of control subjects and patients with HIV

Variables (N (%) or mean ± SD)	Control (*N* = 80)	HIV (*N* = 80)	*P*-value
Demographic findings			
Age (years)	39 ± 8	39 ± 8	0.693
BMI (kg/m^2^)	24.9 ± 3.3	22.9 ± 3.0	< 0.001
Currently smoking	44 (55)	36 (45.0)	0.081
Alcohol ≥3 units/day	3 (4)	3 (4)	1.000
Previous fracture	1 (1)	10 (12.5)	0.009
Underlying disease			
Diabetes mellitus	0 (0)	3 (3.8)	0.245
Hypertension	0 (0)	4 (5.0)	0.120
Dyslipidemia	0 (0)	8 (10.0)	0.028
Laboratory findings			
Urea nitrogen (mg/dl)	13.9 ± 2.8	13.2 ± 3.5	0.154
Creatinine (mg/dl)	0.9 ± 0.1	0.9 ± 0.1	0.928
Calcium (mg/dl)	9.1 ± 0.8	9.1 ± 0.3	0.470
Phosphorus (mg/dl)	3.6 ± 1.5	3.3 ± 0.5	0.130
ESR (mm/h)	7.4 ± 5.4	9.6 ± 9.0	0.081
Hemoglobin (g/dl)	15.6 ± 1.1	15.3 ± 1.0	0.041
Platelet (10^3^/μl)	245 ± 59	228 ± 39	0.033
HIV-specific factors			
Duration since HIV diagnosis (days)		2622.9 ± 1814.9	
Nadir CD4 T-cell counts (cells/μl)		349 ± 183	
Current CD4 count (cells/μl)		669 ± 271	
Current proportion with plasma HIV-1 RNA viral load < 40 copies/ml		75 (93.7%)	
On ART		78 (97.5%)	
On TDF		40 (50.0%)	
Current ART			
NNRTI-based		28	
PI-based		29	
Integrase inhibitor-based		21	
Duration of ART (days)		677.9 ± 411.2	

*SD* Standard deviation, *BMI* Body mass index, *ESR* Erythrocyte sedimentation rate, *ART* Antiretroviral therapy, *TDF* Tenofovir disoproxil fumarate, *NNRTI* Non-nucleoside reverse transcriptase inhibitors, *PI* Protease inhibitor

Among HIV-infected patients, the current mean CD4 T-cell count was 669 ± 271 cells/μl and the nadir CD4 T-cell count was 349 ± 183 cells/μl. Seventy-eight patients (97.5%) were receiving ART, and 93.7% had plasma HIV-1 RNA < 40 copies/ml. The mean duration of ART was 677.9 ± 411.2 days. The ART regimens used were non-nucleoside reverse transcriptase inhibitor (NNRTI)-based (*n* = 28, 35.8%), protease inhibitor (PI)-based (*n* = 29, 37.1%), or integrase inhibitor-based (*n* = 21, 26.9%). Forty-one (51.3%) patients had had exposure to tenofovir disoproxil fumarate (TDF).

### TBS and BMD results

**Fig. 1:**
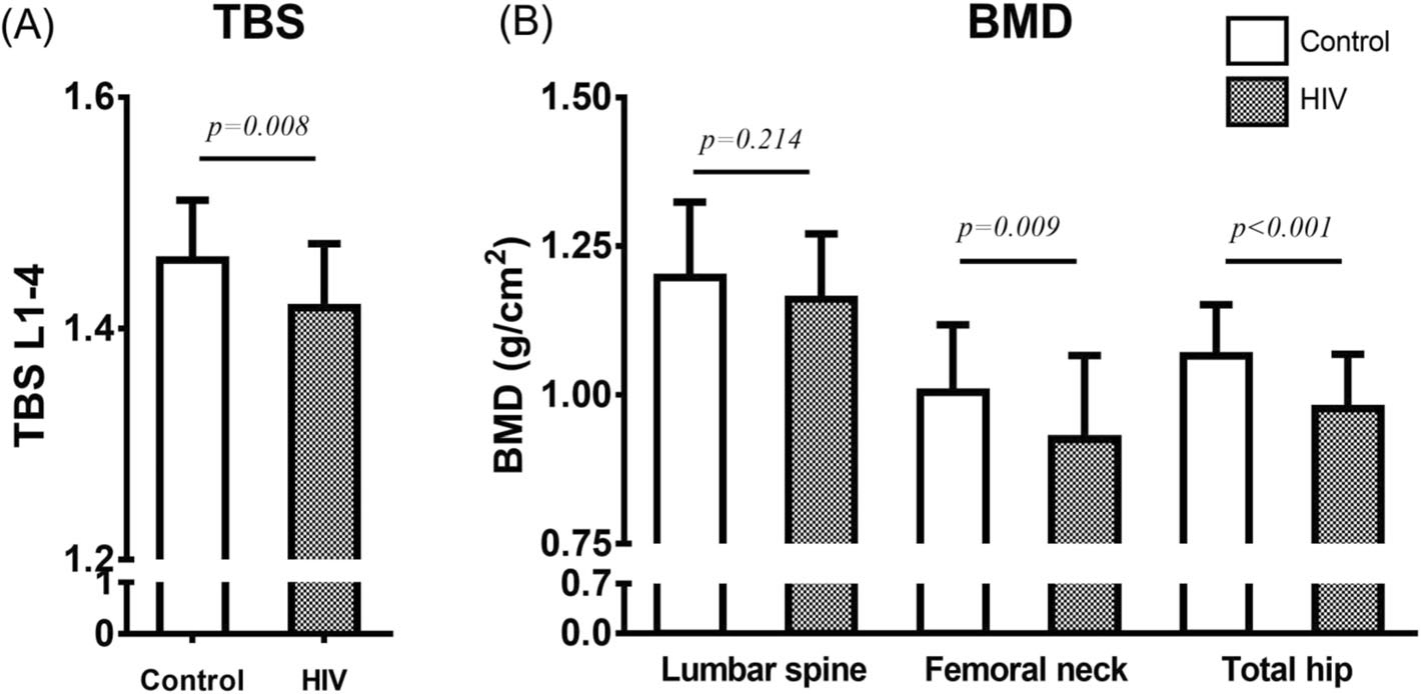
Trabecular bone score (TBS) and bone mineral density (BMD) in HIV-infected male patients and matched controls. **a** Comparison of TBS between male HIV patients and controls. **b** Comparison of BMD at the lumbar spine, femoral neck, and total hip between male HIV patients and controls

In the HIV group, four (5.0%) patients had an intermediate TBS fracture risk and two (2.5%) patients had a high TBS fracture risk; in the matched control group, seven (8.8%) subjects had an intermediate TBS fracture risk. The fracture risk assessed via TBS was not different between the HIV and control groups (*p* = 0.244). However, the mean TBS value in HIV-infected patients was 1.41 ± 0.07, which was significantly lower than the mean value for the matched control group (1.45 ± 0.07, *p* = 0.008) (Fig. 1a).

The proportion of subjects with low BMD was significantly higher in the HIV group than in the control group (21.3% [17/80] vs. 8.8% [7/80], *p* = 0.027). The proportion of subjects with low BMD at the lumbar spine was significantly different between the two groups (21.3% [17/80] vs. 6.3% [5/80], *p* = 0.006), but the proportion with low BMD at either the femoral neck or total hip did not differ between the groups (0% [0/80] vs. 2.5% [2/80], *p* = 0.155). Figure 1b shows the BMD values for the lumbar spine, femoral neck, and total hip for the HIV group and the matched control group. The mean femoral neck BMD values (0.95 ± 0.14 vs. 1.02 ± 0.14, *p* = 0.009) and total hip BMD values (0.98 ± 0.13 vs. 1.06 ± 0.14, *p* < 0.001) were significantly lower in the HIV group than in the matched control group; however, BMD values for the lumbar spine did not differ between the two groups (1.16 ± 0.16 vs. 1.19 ± 0.18, *p* = 0.214).

There was discordance between the BMD and TBS results in 11 patients in the HIV group and 13 subjects in the control group; these proportions were not significantly different (*p* = 0.66).

In both groups, TBS values were positively correlated with BMD at the lumbar spine, femoral neck, and total hip. However, TBS values were not correlated with BMI in either group (Table 2). In the HIV group, TBS values were negatively correlated with the duration of tenofovir exposure (*p* = 0.04) and trended toward a negative correlation with the duration of HIV diagnosis (*p* = 0.07) (Table 2, Additional file 1: Figure S1).

**Table 2:** Correlations between clinical data, BMD and TBS in patients with HIV and matched controls

a. Correlations between BMD and TBS in patients with HIV and matched controls								
Group		BMD, g/cm^2^						
		Lumbar spine		Femoral neck		Total hip		
Control								
TBS, L1–L4		0.449 (< 0.001)		0.393 (< 0.001)		0.446 (< 0.001)		
HIV								
TBS, L1–L4		0.368 (0.001)		0.384 (< 0.001)		0.401 (< 0.001)		
b. Correlations between clinical data and TBS in patients with HIV								
Group	BMI, kg/m^2^	25[OH]vitamin D	C-peptide	Osteocalcin	Duration after HIV diagnosis	Nadir CD4 count	Duration of ART	Duration^a^ of tenofovir exposure
TBS, L1–L4	0.112 (0.321)	0.184 (0.107)	−0.008 (0.946)	−0.088 (0.449)	−0.202 (0.07)	0.031 (0.399)	−0.07 (0.538)	− 0.326 (0.04)

Data are expressed as r coefficients (*p*-value)

*TBS* Trabecular bone score, *BMD* Bone mineral density, *BMI* Body mass index, *ESR* Erythrocyte sedimentation rate

^a^This was displayed as a graph at supplement

### Laboratory tests of bone metabolism in HIV-infected patients

In the group of 80 HIV-infected male patients, the mean 25[OH]D level was 21.2 ± 8.08 ng/ml, with 45% of patients (*n* = 36) measuring below 20 ng/ml. The quartile distribution of 25[OH]D values in patients was as follows: < 10 ng/ml (2.6%), 10–19.99 (43.6%), 20–29.99 (44.8%), 30–39.99 (3.8%), ≥40 (5.1%). Mean calcium, phosphorus, and alkaline phosphatase levels were 9.14 ± 0.32 mg/dl, 3.33 ± 0.51 mg/dl, and 64.7 ± 17.95 U/l, respectively. Mean osteocalcin and CTX levels were 18.91 ± 8.4 μg/ml and 0.39 ± 0.22 μg/ml, respectively.

### Relationship between clinical characteristics and TBS in HIV-infected patients

Table 3 shows the demographic, laboratory, and disease-related parameters in HIV-infected patients with normal TBS values and HIV-infected patients with low TBS values. BMI, current smoking status, and presence of diabetes mellitus did not differ between the normal-TBS and low-TBS groups. Serum creatinine levels were significantly lower in HIV-infected patients with low TBS (*p* = 0.003); however, calcium (*p* = 0.21), phosphorus (*p* = 0.19), alkaline phosphatase (*p* = 0.38), 25[OH]D (*p* = 0.80), osteocalcin (*p* = 0.41), and CTX (*p* = 0.08) levels did not differ between the groups. Duration since HIV diagnosis, nadir CD4 T-cell count, TDF exposure, duration of TDF treatment, duration of ART, and ART regimen type did not differ between the normal-TBS and low-TBS groups.

**Table 3:** Demographic, laboratory, and disease-related variables in HIV-infected patients with normal TBS and low TBS

Variables (N (%) or mean ± SD)	Normal TBS (< 1.31, *N* = 74)	Low TBS (≤1.31, *N* = 6)	*P*-value
Demographic findings			
Age, years	38.4 ± 8.7	37.7 ± 7.6	0.83
BMI (kg/m^2^)	22.9 ± 2.8	23.4 ± 5.5	0.84
Currently smoking	34 (45.9%)	2 (33.3%)	0.550
Alcohol ≥3 units/day	3 (4.1%)	0 (0%)	0.615
Previous fracture	8 (10.8%)	2 (33.3%)	0.109
Underlying disease			
Diabetes mellitus	3 (4.1%)	0 (0%)	0.615
Hypertension	4 (5.4%)	0 (0%)	0.559
Dyslipidemia	8 (10.8%)	0 (0%)	0.395
Laboratory findings			
Total lymphocyte	2921.7 ± 1481.3	2393.8 ± 660.5	0.39
Urea nitrogen (mg/dl)	13.4 ± 3.5	10.9 ± 2.5	0.10
Creatinine (mg/dl)	0.9 ± 0.1	0.77 ± 0.08	0.003
Calcium (mg/dl)	9.1 ± 0.3	9.3 ± 0.2	0.21
Phosphorus (mg/dl)	3.3 ± 0.5	3.6 ± 0.47	0.19
ESR (mm/h)	9.3 ± 9.1	12.1 ± 6.8	0.46
Hemoglobin (g/dl)	15.3 ± 1.0	14.6 ± 0.4	0.12
Platelet (10^3^/μl)	228 ± 40	227 ± 30	0.97
Alkaline phosphatase (U/l)	65.2 ± 18.2	58.5 ± 14.8	0.38
25[OH]D (ng/ml)	21.2 ± 8.2	20.4 ± 6.1	0.80
C-peptide (μg/ml)	0.4 ± 0.2	0.23 ± 0.07	0.08
Osteocalcin (μg/ml)	19.1 ± 8.6	16.2 ± 4.3	0.41
HIV-specific factors			
Duration since HIV diagnosis (days)	2665.2 ± 1827.9	2101.2 ± 1704.1	0.47
Nadir CD4 T-cell counts (cells/μl)	273.3 ± 158.2	360.0 ± 135.2	0.77
RNA level (copies/ml)	151,420.4 ± 380,279.0	27,523.1 ± 54,226.9	0.37
Current CD4 count (cells/μl)	668.2 ± 280.7	679.3 ± 130.0	0.92
Current proportion with plasma HIV-1 RNA levels < 40 copies/ml	69 (93.2%)	6 (100%)	0.511
On ART	73 (98.6%)	5 (83.3%)	0.02
NNRTI	27 (36.5%)	1 (16.7%)	
Protease inhibitor	27 (36.5%)	1 (16.7%)	
Integrase inhibitor	18 (24.3%)	3 (50%)	
On TDF	37 (50.0%)	3 (50.0%)	1.00
Duration of ART by regimen type, days			
NRTI	1964.9 ± 1550.6	1097.7 ± 1156.1	0.18
NNRTI	1304.5 ± 1217.0	570.0 ± 476.5	0.40
PI	1234.5 ± 1479.9	870.6 ± 1195.8	0.59
Integrase inhibitor	155.4 ± 425.8	181.5 ± 271.4	0.88
TDF	671.2 ± 429.2	709.7 ± 226.9	0.89

*SD* Standard deviation, *BMI* Body mass index, *ESR* Erythrocyte sedimentation rate, *ART* Anti-retroviral therapy, *TDF* Tenofovir disoproxil fumarate, *NRTI* Nucleoside reverse transcriptase inhibitors, *NNRTI* Non-nucleoside reverse transcriptase inhibitors, *PI* Protease inhibitor

## Discussion

In this cross-sectional study, TBS values were significantly lower in HIV-infected young male patients than in age- and sex-matched controls. Furthermore, TBS values were positively correlated with BMD at the lumbar spine, femoral neck, and total hip and negatively correlated with the duration of TDF exposure.

The difference in TBS values between individuals with and without HIV is consistent with prior results from the Women’s Interagency HIV Study [14]. It is well known that age, postmenopausal status, smoking, and low BMI are risk factors for osteoporosis in the general population, and that HIV-related factors such as the direct action of the virus, chronic immune activation, and antiviral toxicity can also affect bone structure [2, 12, 15, 16]. A strength of the present study is that we included only HIV-infected male patients aged between 18 and 50 years to minimize the effects of the general risk factors.

TDF, an acyclic nucleotide analog of adenosine monophosphate, is widely used as a core component of many ART regimens, and several longitudinal studies have reported an association between TDF treatment and significantly reduced BMD [17, 18]. Thus, long-term use of TDF may lead to clinically relevant changes in BMD. One study reported a significant decrease in BMD and TBS after 1 year of TDF therapy [19]. We found that TBS, a measure of bone microarchitecture, was negatively correlated with TDF exposure, suggesting that TDF can affect not only the quantity of bone, but also its microarchitecture. We did not analyze the factors associated with low TBS because of the small number of patients with low TBS values; however, creatinine levels differed between the low-TBS and normal-TBS patient groups. Although the mechanism by which TDF causes bone toxicity is still unclear, TDF has both direct and indirect effects on bone via renal and endocrine systems [18, 20]. Subclinical tubulopathy may be a key factor in TDF-driven reductions in BMD, and our results support the hypothesis that renal function may affect bone microarchitecture in HIV-infected patients. This study also shows that TBS was marginally negatively correlated with the duration of HIV diagnosis, and uncorrelated with serum markers of bone turnover. CTX levels were slightly lower in the low-TBS patient group. HIV infection decreases bone formation and increases bone loss through direct effects related to the virus as well as through indirect effects related to pro-inflammatory cytokines, resulting in an increase in bone resorption and loss. However, the pathophysiology of bone fragility with HIV infection remains incompletely understood [2, 21]. Our results support the hypothesis that virus-associated factors, in addition to a patient’s clinical risk factors, contribute to bone turnover in young HIV-infected male patients despite suppression of the virus by ART. Animal experiments have shown that low BMI leads to osteoclast activation through enhanced production of the receptor activator of nuclear factor kappa-B (RANK) ligand by B-cells, accompanied by downregulation of the antagonist osteoprotegerin [22]. In the present study, the HIV-infected patients had lower BMI scores than the control subjects, which may have affected the bone. Further studies with larger patient groups are warranted to assess the risk factors and bone turnover markers associated with low TBS.

This study found that 21.3% of young male HIV-infected patients had a low BMD for their age (Z-score ≤ − 2.0). Currently, screening for osteoporosis with DXA is recommended in male HIV patients aged ≥50 years [12, 23]. A previous meta-analysis found that 67% of 884 HIV-infected patients had reduced BMD, of whom 15% had osteoporosis, yielding pooled odds ratios of 6.4 and 3.7, respectively, compared with non-HIV-infected controls [24], similar to our results for HIV-infected men aged 50 years and younger. Therefore, our results question the current guidelines for DXA screening in young HIV-infected men, although further studies will be needed to confirm our results. In this study, 7.5% of young HIV-infected men were deemed at high risk of fracture according to the TBS value, although a similar percentage was found for the control group. TBS, a texture index derived from DXA of the lumbar spine, is a widely available and endorsed technique and is included in the World Health Organization (WHO) Fracture Risk Assessment Tool [25, 26]. The correlation coefficient between TBS and BMD varies depending on the study. Some studies have shown that TBS correlates with lumbar BMD; however other studies reported that TBS correlates poorly with BMD [8, 27, 28]. This may be due to the diversity in the underlying diseases and differences in the demographic characteristics of the participants. Studies on the role of TBS as a complement to BMD and as a means of identifying cut-off values to predict fracture risk in HIV-infected populations are limited. Thus, additional studies are warranted to clarify the role that TBS may play in this patient population.

In this study, BMD values of the lumbar spine were not statistically significant between the two groups, although BMD results in HIV-infected patients were lower than in controls. A meta-analysis showed that the prevalence of osteopenia/osteoporosis of the lumbar spine [OR = 2.4 (95% Cl: 2.0–2.8)] and hip [OR = 2.6 (95%Cl: 2.2–3.0)] was significantly higher in HIV-infected groups than in controls [1]. This is probably due to sampling bias or the small number of patients. The pathogenesis of bone loss in HIV-infected patients is a complex and has not been established. Therefore, there exists a possibility that unknown processes are involved in bone turnover in HIV-infected patients.

This study has several limitations. First, laboratory markers related to bone metabolism, such as vitamin D and osteocalcin, were not examined in the control group so we could not compare these parameters between the two groups. Second, we did not obtain long-term follow-up TBS data, and were therefore unable to assess the extent to which TBS predicts fractures. Third, sampling bias could have occurred in the HIV-infected patients group and the control group.

## Conclusion

We have presented a cross-sectional study of HIV-infected men aged 50 years and younger, and assessed bone microarchitecture via TBS in this patient population. We observed lower TBS and BMD values in HIV-infected young men than in the control group and found that TBS values were positively correlated with BMD at the lumbar spine, femoral neck, and total hip, and negatively correlated with the duration of TDF exposure.

## Supplementary information

**Additional file 1: Figure S1.** Correlation between trabecular bone score (TBS) and TDF exposure time. TDF, Tenofovir disoproxil fumarate.

## Data Availability

Not applicable.

## Abbreviations

25[OH]D: 25-hydroxy vitamin D
ART: Antiretroviral therapy
BMD: Bone mineral density
BMI: Low body mass index
CTX: C-terminal cross-linking telopeptide of type I collagen
DXA: Dual-energy X-ray absorptiometry
PTH: Parathyroid hormone
SDs: Standard deviations
TBS: Trabecular bone score
TDF: Tenofovir disoproxil fumarate
