# Supplementary figures and images for: Trabecular bone scores in young HIV-infected men: a matched case-control study
Source: BMC Musculoskelet Disord. 2020 Feb 10;21:94. doi: 10.1186/s12891-020-3092-0 (PMC7011600; doi:10.1186/s12891-020-3092-0)

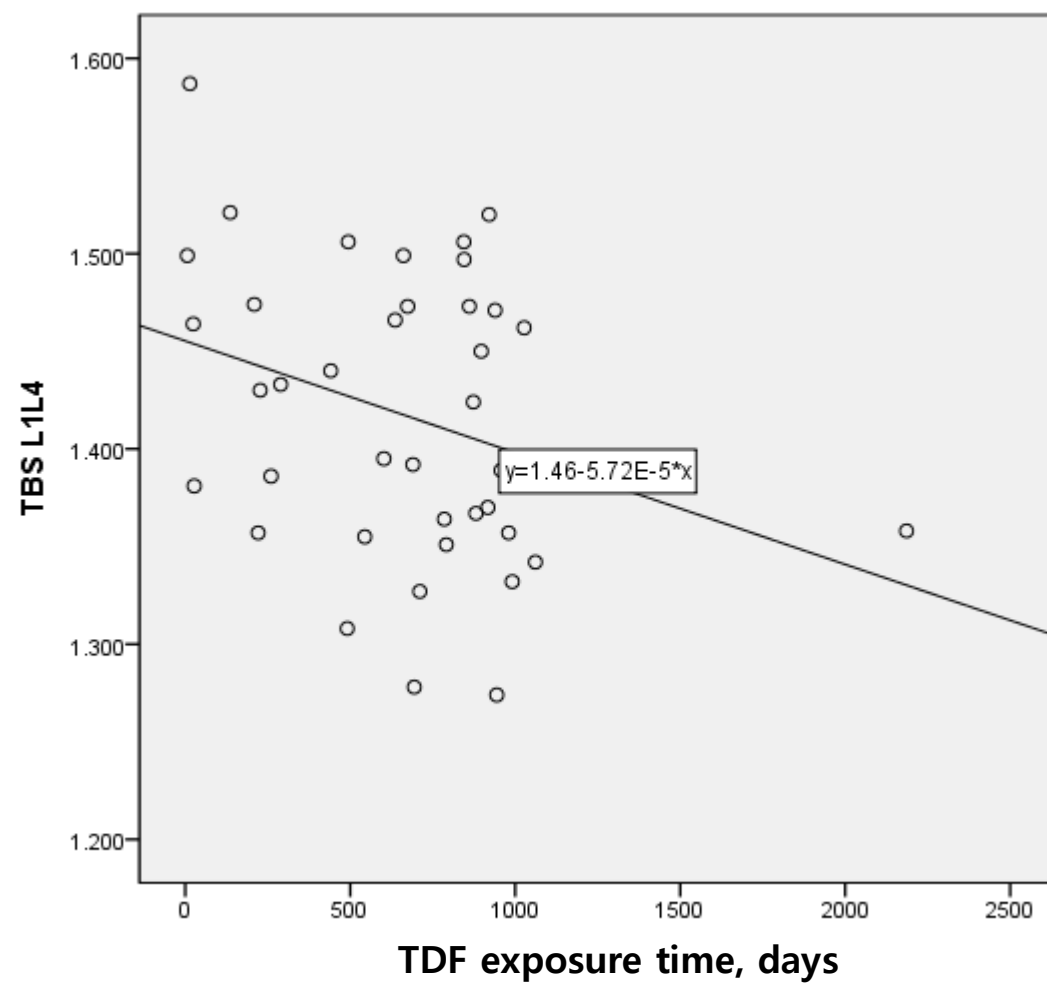

Supplement: Supplementary file 1 — Additional file 1: Figure S1. Correlation between trabecular bone score (TBS) and TDF exposure time. TDF, Tenofovir disoproxil fumarate. [file 12891_2020_3092_MOESM1_ESM.pdf]
